# Computer-Aided Modelling and Analysis of PV Systems: A Comparative Study

**DOI:** 10.1155/2014/101056

**Published:** 2014-01-02

**Authors:** Charalambos Koukouvaos, Dionisis Kandris, Maria Samarakou

**Affiliations:** ^1^School of Engineering and Physical Sciences, Heriot Watt University, Edinburgh EH14 1AS, UK; ^2^Central Greece Energy Distribution Administration, Public Power Corporation S.A., 34500 Aliveri, Greece; ^3^Department of Electronic Engineering, School of Technological Applications, Technological Educational Institute of Athens, 12210 Athens, Greece; ^4^Department of Energy Technology Engineering, School of Technological Applications, Technological Educational Institute of Athens, 12210 Athens, Greece

## Abstract

Modern scientific advances have enabled remarkable efficacy for photovoltaic systems with regard to the exploitation of solar energy, boosting them into having a rapidly growing position among the systems developed for the production of renewable energy. However, in many cases the design, analysis, and control of photovoltaic systems are tasks which are quite complex and thus difficult to be carried out. In order to cope with this kind of problems, appropriate software tools have been developed either as standalone products or parts of general purpose software platforms used to model and simulate the generation, transmission, and distribution of solar energy. The utilization of this kind of software tools may be extremely helpful to the successful performance evaluation of energy systems with maximum accuracy and minimum cost in time and effort. The work presented in this paper aims on a first level at the performance analysis of various configurations of photovoltaic systems through computer-aided modelling. On a second level, it provides a comparative evaluation of the credibility of two of the most advanced graphical programming environments, namely, Simulink and LabVIEW, with regard to their application in photovoltaic systems.

## 1. Introduction

Serious economic concerns along with the growing worries about the disastrous effects that technological progress accumulatively causes to world environment, impose a shift towards renewable energy resources, which constitute an environmental friendly approach for power generation with satisfactory efficiency [1]. That is why in many regions of the globe there is an official tendency to let renewable energy sources have an increasing share in the overall power production process. For instance, European Union, by means of the implementation of the so-called 20/20/20 initiative, aims to achieve by year 2020 not only the reduction of greenhouse gas emissions by 20% (compared to 1990 ones), but also the increase of the magnitude of renewable energy to 20% and the decrease of the overall energy consumption by 20% through efficient energy management [2].

The renewable energy resources which have the most enhanced exploitation, thanks to relevant advances in modern scientific research, are solar, hydropower, geothermal, biomass, and wind energy [3, 4].

Solar energy refers to the solar radiation that reaches the Earth and can be converted into other forms of energy, such as heat and electricity. The transformation of solar radiation into electric current is performed by using photovoltaic (PV) cells. A PV cell is practically a p-n junction placed in the interior of a thin wafer of semiconductor. The solar radiation falling on a PV cell can be diconverted to electricity through the so-called photovoltaic effect. This is a physical phenomenon in which photons of light excite electrons into higher energy states letting them act as charge carriers for electric current. Specifically, the exposure of a PV to sunlight triggers the creation of electron-hole pairs proportional to the incident irradiation by photons having energy greater than the band-gap energy of the semiconductor material of the PV cell.

Over the last decades, the international interest in the PV conversion of solar radiation is continuously growing. In this way, the use of PV systems is nowadays widespread to an extent that is considered to constitute the third greater renewable energy source in terms of globally installed capacity, after hydro- and wind power.

On the other hand, the brilliant prospects of PV systems for further evolution get obstructed due to various technical and economic issues that have yet to be resolved. For this reason modern scientific and technological research focuses on the development of methodologies and equipment for the increase of energy efficiency of PV systems, the reduction of their production cost, the improvement of their market penetration, and the enhancement of their environmental performance [5–7].

These research activities can be greatly assisted by the utilization of software tools for the development of models of the PV systems under consideration and the analysis of their performance by carrying out simulation tests.

The aim of this paper is to perform the computer-aided design and performance analysis of both grid-connected and standalone PV systems by using in parallel the two most widely used graphical programming environments, namely, Simulink by Mathworks and LabVIEW by National Instruments. This task is accomplished by taking advantage of the potential provided through the adaptation of these two software platforms to systems of PV nature. The parallel utilization of MATLAB and LabVIEW enables the comparative evaluation of their accuracy, validity, and their overall performance under various simulation scenarios. According to the knowledge of the authors of this paper, this is the first comparative study of this kind which is performed in the field of photovoltaics, although similar studies have been carried out in other scientific areas such as [8].

The rest of the paper is organized as follows: Section 2 focuses on related work in the computer-assisted modelling of PV systems.  Section 3 describes the procedures used for the PV systems modelling. In Section 4 the results of the simulation tests executed are presented. The discussion of these results is performed in Section 5. Finally, Section 6 concludes the paper.

## 2. Computer-Aided Performance Analysis of PV Systems

The performance of PV plant is subject to many parameters, the influence of which should be accurately estimated before any investment is made for the establishment of this plant. Actually, the temperature and solar radiation are the two main factors affecting the performance of PV systems. Specifically, ambient temperature should definitely be taken into consideration because the relationship between the temperature and the efficiency of solar cells is inversely proportional [9]. Additionally, the energy produced by a PV system on an annual basis is directly related to the available solar radiation and therefore depends on the geographical location of the system because two radiation beams of equal power but of different wavelength can produce different amounts of electricity in a solar cell and thus form a different degree of performance [9]. Moreover, the operational efficiency of a solar panel may be affected by other environmental or other type factors such as the speed and direction of wind, the distribution of the solar spectrum, rain, shading, pollution, panel aging, optical losses, and panel casualties [9–13].

In order to calculate the energy efficiency of PV systems in an accurate and systematic modern research works focus on the computer-aided design and analysis of this type of systems [14].

For instance, a PV array simulation model incorporated in Simulink GUI environment was developed using basic circuit equations of the PV solar cells including the effects of solar irradiation and temperature changes. The developed model was tested by means of both a directly coupled DC load and an AC load via an inverter [15].

In another work a model for a solar PV array (PV), with an AC/DC converter and load, a maximum power point tracker, a battery, and a charger, was built. The whole model was simulated under four testing scenarios some of which included cloudy and sunny conditions along with constant and varying load [16].

Similarly, an approach to determine the characteristic of a particular PV cell panel and to study the influence of different values of solar radiation at different temperatures concerning performance of PV cells in Simulink was proposed [17]. There are research works, such as [18], which focus on modelling and simulation of standalone PV systems, whilst others, like [19], address modelling and simulation of grid-connected PV systems to analyze the grid interface behavior and control performance in the system design. Other works, such as [20], predict energy production from PV panels under various performance conditions.

In [21] a generalized PV model built in MATLAB/Simulink, which enables the simulation of the system dynamics, is presented. Similarly, [22] focuses on the modelling process of PV cells in Simulink while in [23] the two-diode model of PV module is examined.

Concentrating on educational applications, [24] describes the design and implementation of a virtual laboratory for PV power systems based on the Cadence PSpice circuit simulator. In [25] an integrated PV monitoring system having a graphical user interface developed in LabVIEW is presented.

## 3. Theoretical Background

The procedure of building accurate PV models is extremely important in order to achieve high efficiency in the decision making related to establishment and operation of PV systems. This justifies why, as already discussed in Section 2, many research works are carried out in this scientific area.

**Figure 1:**
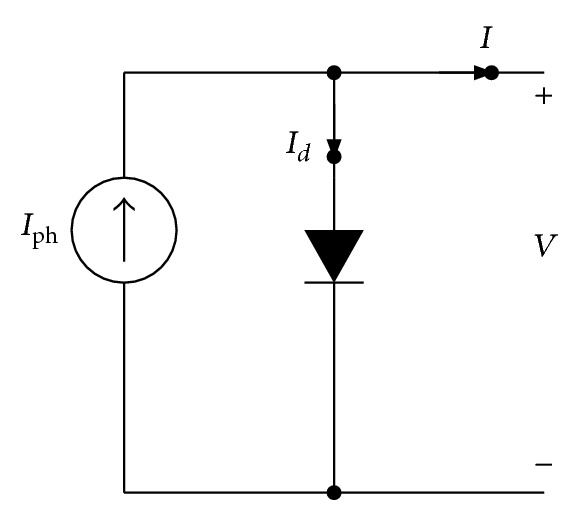
Electronic circuit equivalent to the model of an ideal PV cell.

Generally, modelling of PV systems is based on the assumption that the operation of PV cells may be simulated by examining the operation of analogous electronic circuits. The simplest PV model found in bibliography comprises a single diode connected in parallel with a light generated current source (*I*_ph_) as shown in Figure 1, where *I*_*d*_ expresses the diode current [26, 27]. This simplified model refers to an ideal solar cell.

**Figure 2:**
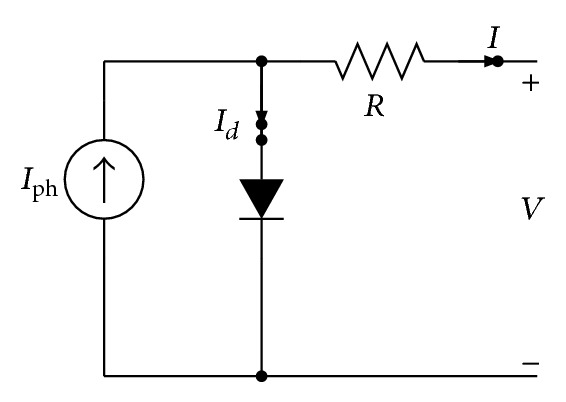
Electronic circuit representing a one-diode with serial resistance model of PV cell.

Going one step further, a series resistance *R*_*s*_, representing an internal parasitic resistance which reduces the efficiency of the solar cell, is added to the circuit, as depicted in Figure 2 [28, 29]. The relationship between the voltage and the current for a given temperature and solar radiation is given as

(1) $$\begin{array}{c} I=I_{\text{ph}}-I_{d}=I_{\text{ph}}-I_{0}\cdot \left(e^{\left(V+I\cdot R_{s}\right)/\left(a\cdot V_{T}\right)}-1\right), \end{array}$$

where *I*_0_ expresses the diode reverse saturation current, *a* represents the ideality factor of the diode, and *V*_*T*_ stands for the so-called thermal voltage of the PV module which is given by [2]

(2) $$\begin{array}{c} V_{T}=\frac{N_{s}\cdot k\cdot T_{c}}{q}, \end{array}$$

where *N*_*s*_ denotes the number of photovoltaic cells connected in series in the PV module, *k* stands for Boltzmann's constant (1.3806503 · 10^−23^ J/K), *T*_*c*_ indicates the temperature of the photovoltaic cell (expressed in Kelvin degrees), and *q* symbolizes the elementary electric charge (1.60217646 · 10^−19^ C).

Other scientific researches make use of a model which is derived by the addition of shunt resistance *R*_*p*_ in parallel to the diode in order to improve model accuracy in the cases of high variations of temperature and low voltage but in expense of complexity [30, 31].

A few research works adopt an ever more complex model, known as the two-diode model aiming to take into consideration recombination losses, that is, the elimination of mobile electrons and electron holes due to the existence of impurities or defects at the front or/and the rear surfaces of a cell. This model includes a second diode placed in parallel to the current source and the initial diode, thus calling for the simultaneous calculation of seven parameters based on either iteration approaches or more analytical methodologies [23].

When simulating a PV module, no matter which model is adopted, the goal is to calculate the *I-V* and *P-V* curves which are representative of the module operation. In this curve the so-called maximum power point (MPP) is determined as the point for which the power dissipated to the load is maximum. From basic circuit theory, the power delivered from or to a device is maximized where the derivative (graphically, the slope) *dI*/*dV* of the *I-V* curve is equal to and opposite of the *I*/*V* ratio (where *dP*/*dV* = 0) and corresponds to the “knee” of the curve.

Another technical feature that is taken into consideration during the simulation tests performed is the so-called total harmonic distortion (THD). Generally, THD of a signal is a measurement of the harmonic distortion present and is defined as the ratio of the sum of the powers of all harmonic components to the power of the fundamental frequency. In energy systems THD is used to characterize their power quality of electric power.

Finally, in order to carry out the comparative performance analysis of the modelling accuracy of Simulink and Labview the so called coefficient of determination was calculated. This is a statistic term denoted as *R* ^2^, which expresses the proportion of total variation of outcomes explained by a model, thus, providing a measure of how well-observed outcomes are replicated by this model.

## 4. Modeling and Simulation Setup

In accordance with the aforementioned, in Section 2, PV modelling studies the research work presented in this paper investigates the computer-aided modelling and analysis of first a standalone commercially available solar panel, second an integrated PV system without grid connection, and third a grid-connected PV system under low and high load conditions.

### 4.1. Modeling of a PV Panel

The first modelling procedure performed refers to the modelling and performance analysis of a single PV panel for the production of *I*-*V* and *P*-*V* curves in maximum power point (MPP). The modelling was performed both in Simulink and LabVIEW based on the aforementioned, in Section 3, one-diode with serial resistance PV model, because it has an adequate ratio of accuracy to complexity.

**Figure 3:**
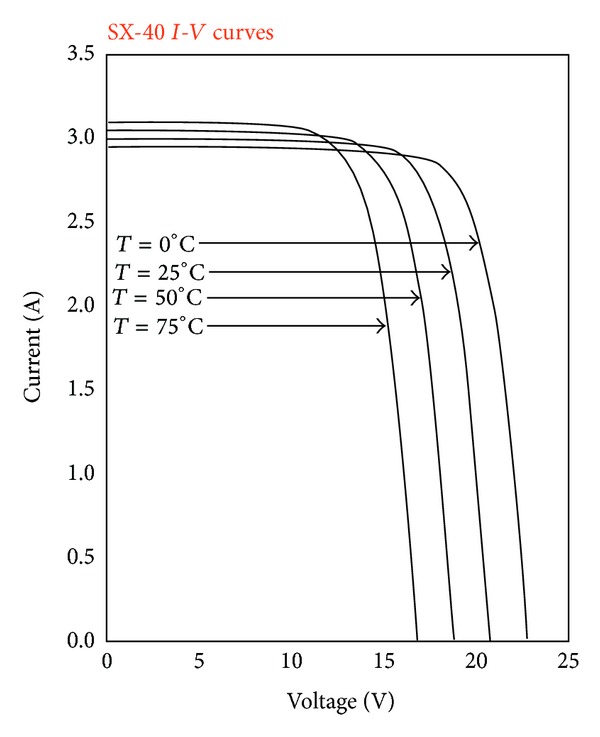
Characteristic *I-V* curve of an SX-50 PV panel.

**Figure 4:**
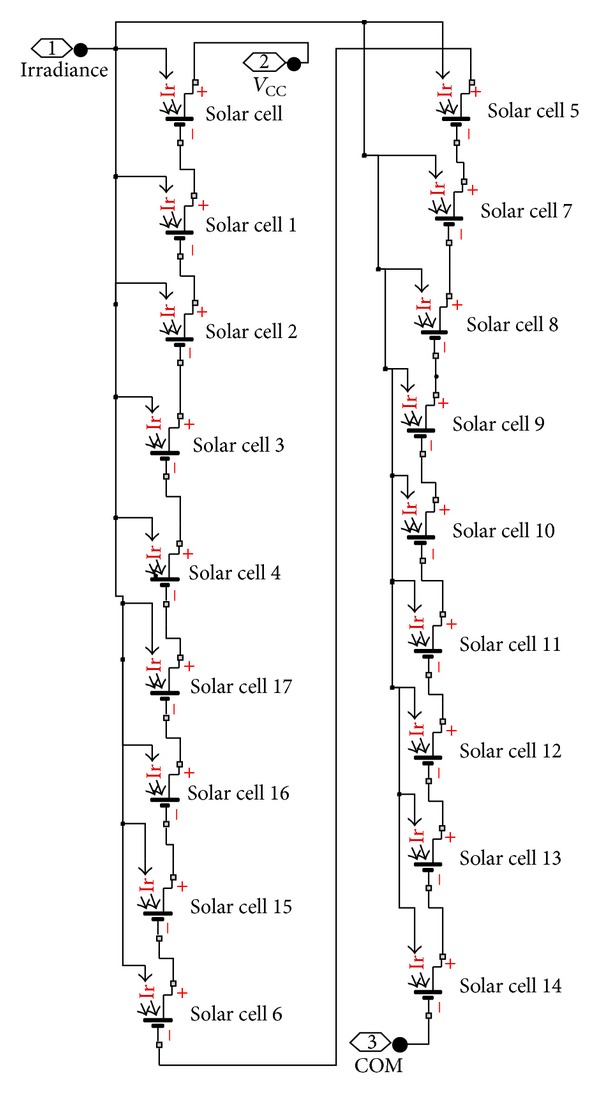
Overview of an SX-50 PV panel in Simulink.

**Figure 5:**
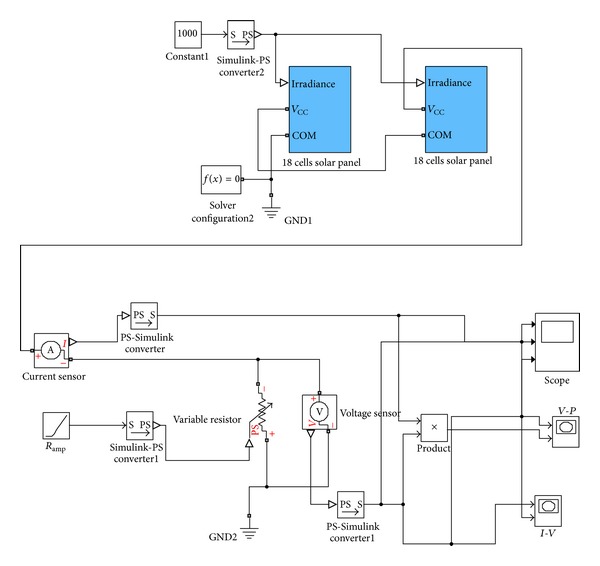
Simulation model of an SX-50 panel in Simulink.

**Figure 6:**
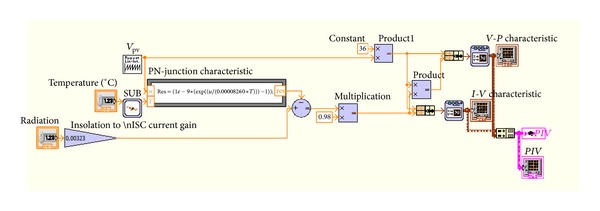
Simulation model of an SX-50 panel in LabVIEW.

The specific PV panel examined is Solarex SX-50. The characteristic features of this PV panel along with their typical values, for solar irradiance *S* = 1000 W/m^2^ and operation temperature *T* = 25°C, are presented in Table 1, while the *I-V* curve for various operating temperatures is illustrated in Figure 3. The specific panel consists of 36 photovoltaic silicon cells located in two rows of 18 PV cells each. The modelling of SX-50 PV panel in Simulink was performed by using Simscape-SimElectronics library.  Figure 4 shows that the structure of the PV panel consisted of two sets of 18 cells each. The corresponding simulation model built is depicted in Figure 5. The modelling of SX-50 PV panel in LabVIEW was performed by using NI LabVIEW Simulation Interface Toolkit.  Figure 6 shows the resultant model developed.

### 4.2. Modeling of an Integrated PV System with No Grid Connection

**Figure 7:**
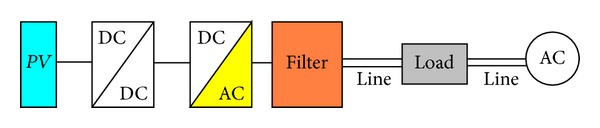
Overview of the integrated PV system modeled.

The second modelling procedure performed refers to the modelling and performance analysis of a typical integrated PV system, consisting of a PV energy source, a DC/DC converter, a DC/AC inverter, a filter, and a load as shown in Figure 7.

The modelling was performed both in Simulink and LabVIEW based on the assumptions that the power of the photovoltaic generator is equal to 4.6 KW and the operation temperature is set to 25°C. Additionally, solar irradiance is supposed to have a stable value equal to 1000 W/m^2^ while the load magnitude is equal to 2 KW. The system was connected to a low-voltage grid by using alternatively a low load (1.5 KW) or a high load (6.5 KW).

**Figure 8:**
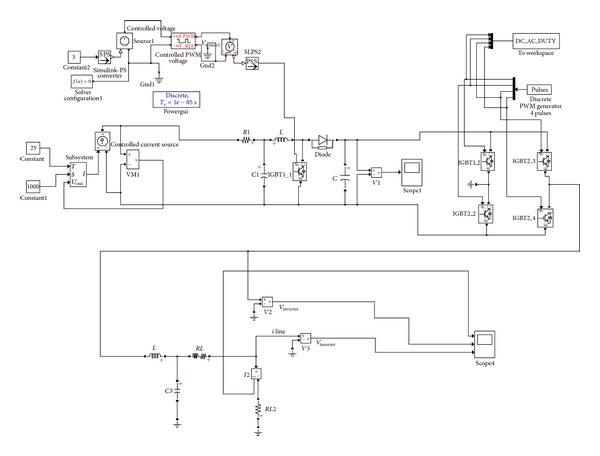
Integrated PV system model in Simulink.

**Figure 9:**
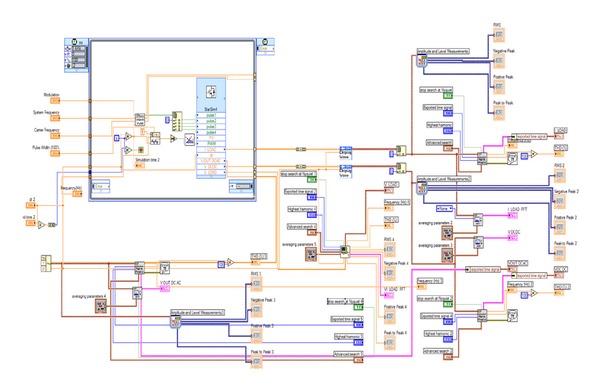
Integrated PV system model in LabVIEW.

The filter is an LC circuit aiming to cut off the range of the output current which produces the DC-AC inverter harmonic frequencies. Similarly, an RL circuit was incorporated in the line that connects the filter with the load.  Figure 8 illustrates the simulation model developed in Simulink while Figure 9 depicts the same model built in LabVIEW.

### 4.3. Modeling of an Integrated PV System with Connection to Low-Voltage Grid

The third modelling task performed refers to the model development and performance analysis of a typical integrated PV system, having the structure shown in Figure 7, which is connected to low-voltage grid by using either a low load (1.5 KW) or high load (6.5 KW).

**Figure 10:**
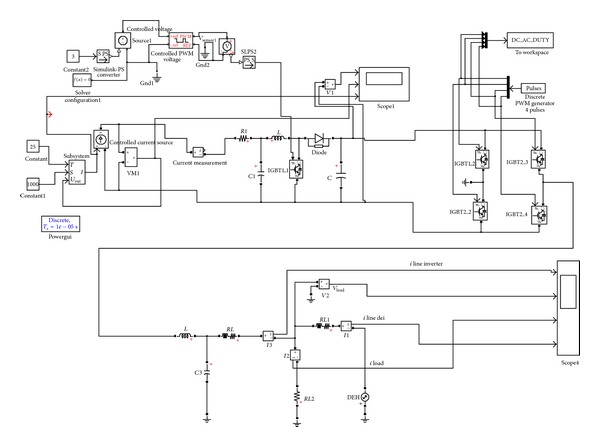
Grid-connected integrated PV system model in Simulink.

**Figure 11:**
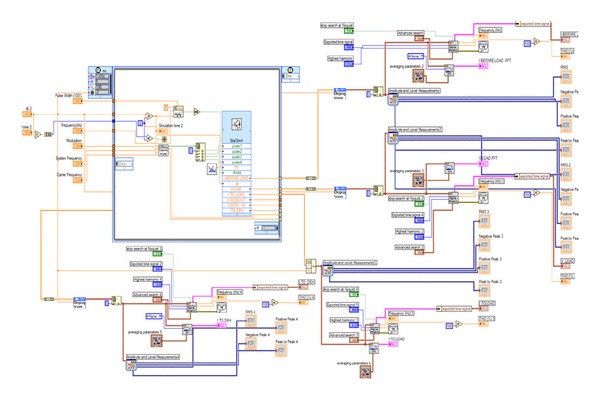
Grid-connected integrated PV system model in LabVIEW.

The filter incorporated in the overall system structure is an LC circuit aiming to cut off the range of the output current which produces the DC-AC inverter harmonic frequencies. Similarly, an RL circuit was used in the line which connects the filter with the load.  Figure 10 illustrates the simulation model developed in Simulink while Figure 11 depicts the same model built in LabVIEW.

## 5. Simulation Results and Discussion

In the following subsections of Section 5 the outcomes of the simulation tests carried out are both described and commented on.

### 5.1. Performance Evaluation of the PV Panel Modeled

**Figure 12:**
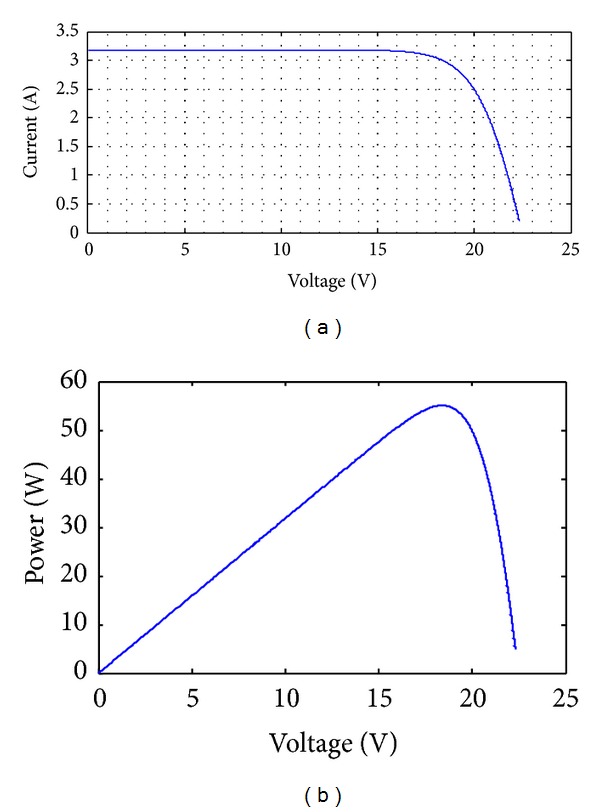
*I*-*V* and *P*-*V* curves for SX-50 Solarex PV panel when *T* = 25°C and *S* = 1000 W/m^2^ in Simulink.

**Figure 13:**
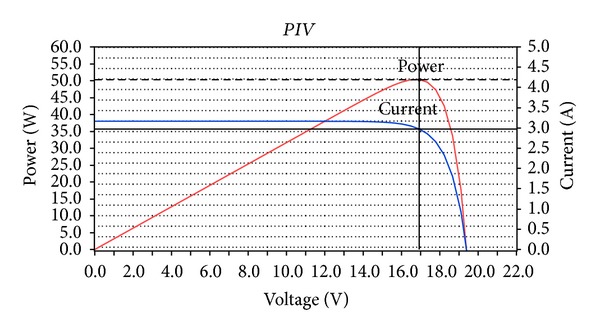
*I*-*V* and *P*-*V* curves for SX-50 Solarex PV panel when *T* = 25°C and *S* = 1000 W/m^2^ in LabVIEW.

The basic assumption in all simulations tests carried out for the performance analysis of the solar panel modeled in Section 4.1 was that there was constant solar irradiance *S* equal to 1000 W/m^2^, while operation temperature *T* was sequentially set to 0°C, 25°C, 50°C, and 75°C. For each one of these temperature values the corresponding *I*-*V* and *P*-*V* curves were drawn and the values of *I*_max⁡⁡_, *V*_max⁡⁡_ and *P*_max⁡⁡_ were calculated both in Simulink and LabVIEW. Indicatively, Figures 12 and 13 depict these curves for *T* = 25°C in Simulink and LabVIEW.

The values of *I*_max⁡⁡_, *V*_max⁡⁡_, and *P*_max⁡⁡_ which were found based on the simulation results in Simulink and LabVIEW for *T* = 0°C are presented in Table 2. The corresponding results for *T* = 50°C are presented in Table 3 and for *T* = 75°C in Table 4.

In a similar way, in Table 5, the values of *I*_max⁡⁡_, *V*_max⁡⁡_, and *P*_max⁡⁡_ calculated through the simulation results by using Simulink and LabVIEW for *T* = 25°C are presented against the corresponding values provided by the manufacturer of the PV panel, that is, Solarex [32].

The percentage deviation of the simulation data of *I*_max⁡⁡_, *V*_max⁡⁡_, and *P*_max⁡⁡_ from the corresponding dfault values provided by Solarex is shown in Table 6.

**Figure 14:**
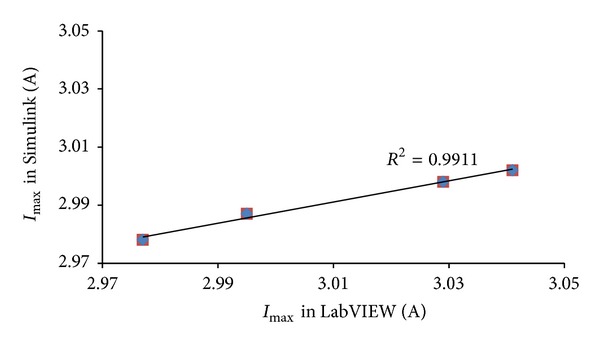
Schematic comparison of the values of *I*_max⁡⁡_ in Simulink and LabVIEW for *T* = 0°C, 25°C, 50°C, and 75°C.

**Figure 15:**
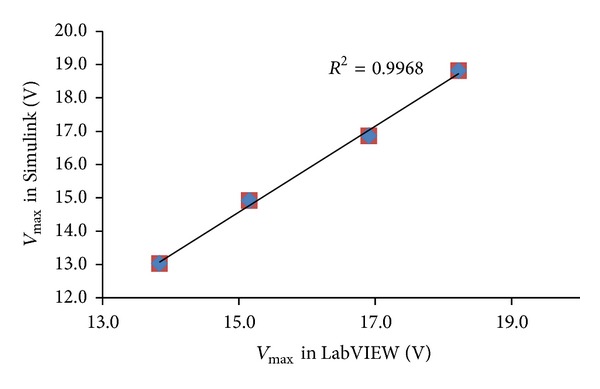
Schematic comparison of the values of *V*_max⁡⁡_ in Simulink and LabVIEW for *T* = 0°C, 25°C, 50°C, and 75°C.

**Figure 16:**
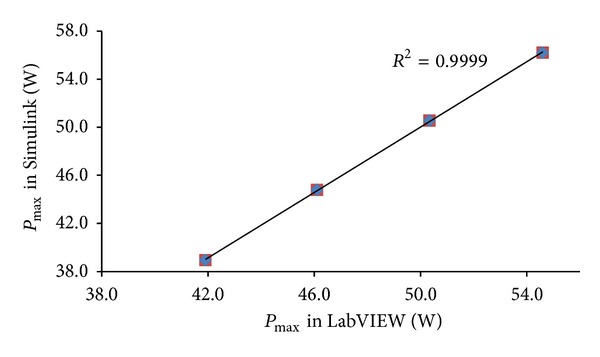
Schematic comparison of the values of *P*_max⁡⁡_ in Simulink and LabVIEW for *T* = 0°C, 25°C, 50°C, and 75°C.

The evaluation of the correlation existing between the simulation results attained alternatively via Simulink and LabVIEW for *I*_max⁡⁡_, *V*_max⁡⁡_, and *P*_max⁡⁡_ is obtained through the calculation of the coefficient of determination (*R* ^2^). As shown in Figures 14, 15, and 16 the values of *R* ^2^ found correspondingly for *I*_max⁡⁡_, *V*_max⁡⁡_, and *P*_max⁡⁡_ are equal to 0.989, 0.996, and 0.999.

### 5.2. Performance Evaluation of an Integrated PV System with No Grid Connection

The performance analysis of the integrated PV described in Section 4.2 was carried out by the investigation of specific characteristics of the system, that is, the system output voltage at load and the current at load by performing Fourier transformation. Simulink makes use of the so-called Powegui FFT Analysis tool which enables the implementation of fast Fourier transformation of signals. Similarly, LabVIEW makes use of STARSIM which is an electrical system simulation tool.

**Figure 17:**
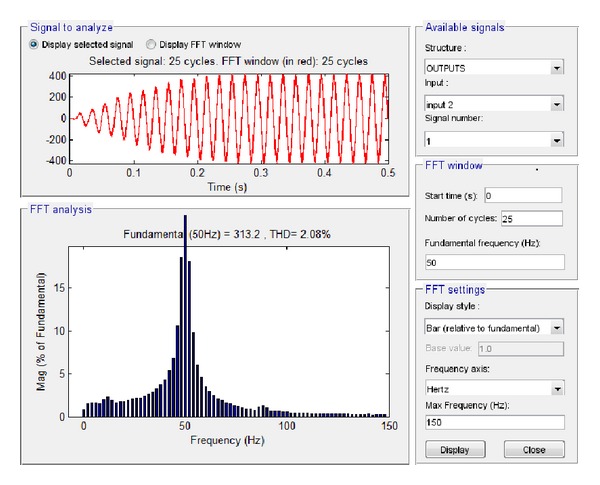
Voltage at load with no grid connection in Simulink.

**Figure 18:**
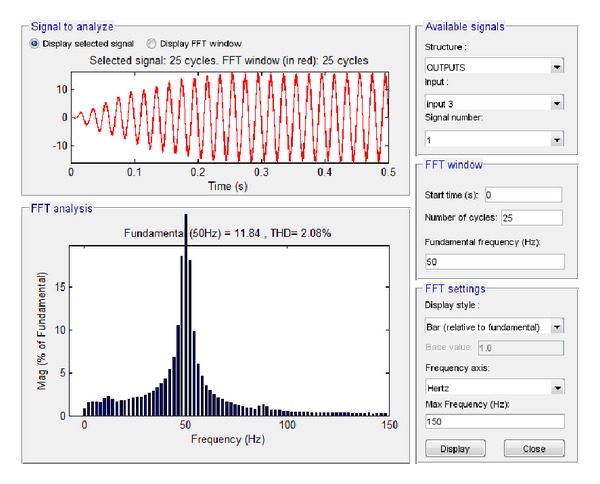
Current at load with no grid connection in Simulink.

The results of the simulation of these two features in Simulink are depicted in Figures 17 and 18.

By applying fast Fourier transformation, it was found that the total harmonic distortion at load is 2.08%, while the amplitude of the voltage at the fundamental frequency is 313.2 V and the amplitude of the current at the fundamental frequency is 11.84 A.

**Figure 19:**
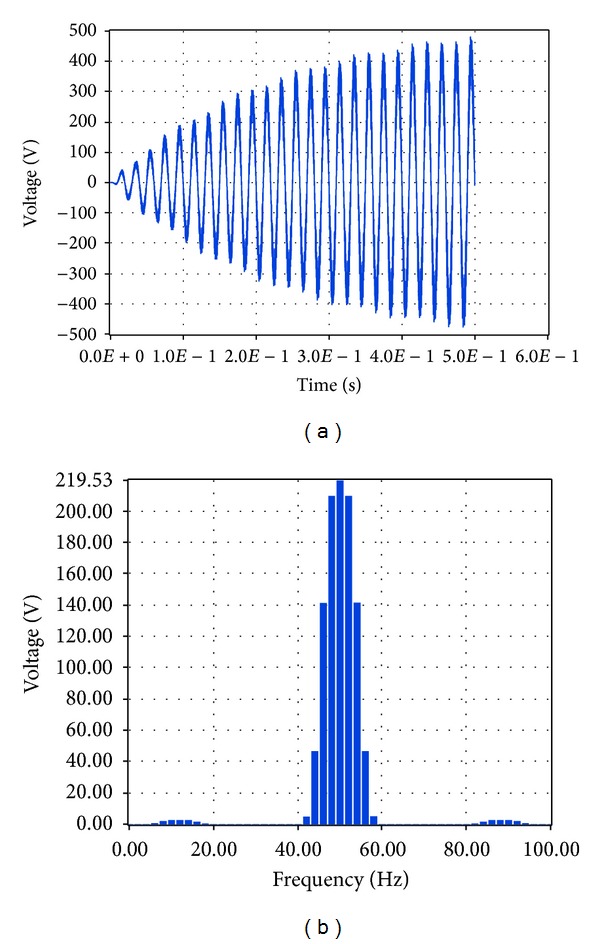
Voltage at load with no grid connection in LabVIEW.

**Figure 20:**
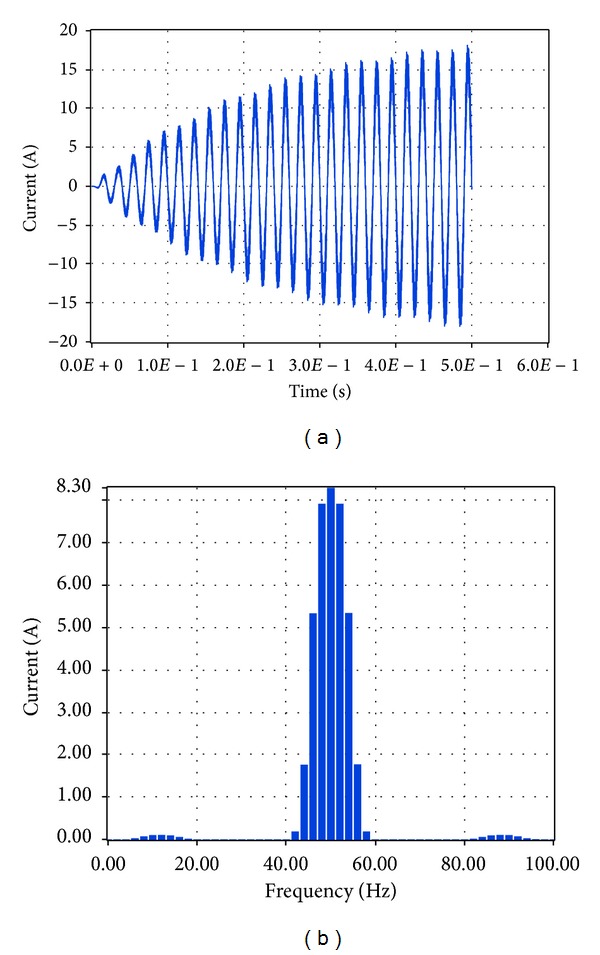
Current at load with no grid connection in LabVIEW.

The corresponding simulation results in LabVIEW proved that the total harmonic distortion at load is 3.78%, while the amplitude of the voltage at the fundamental frequency is 219.53 V RMS, that is, 310.46 V, and the amplitude of the current at the fundamental frequency is 8.3 A RMS, that is, 11.74 A, as shown correspondingly in Figures 19 and 20.

The comparison of these data shows that the simulation through Simulink and LabVIEW leads to results which have a percentage deviation less than 0.9% for both voltage at load and current at load.

### 5.3. Performance Evaluation of an Integrated PV System with Connection to Low-Voltage Grid

By using the same software tools as in the last subsection, the performance analysis of the, described in Section 4.3, integrated PV connected to low-voltage grid was carried out.

**Figure 21:**
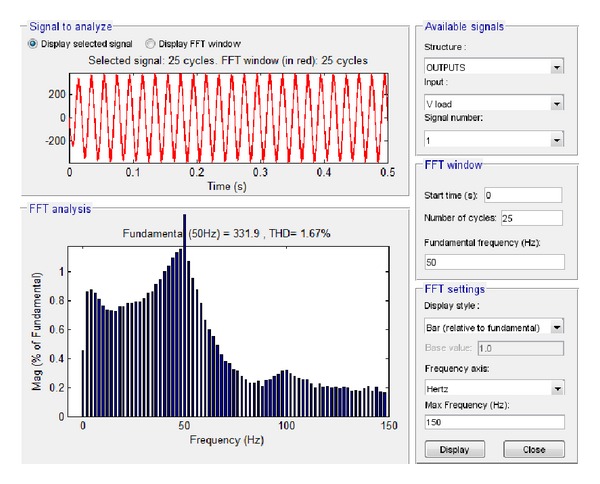
Voltage at load 1.5 KW with grid connection in Simulink.

**Figure 22:**
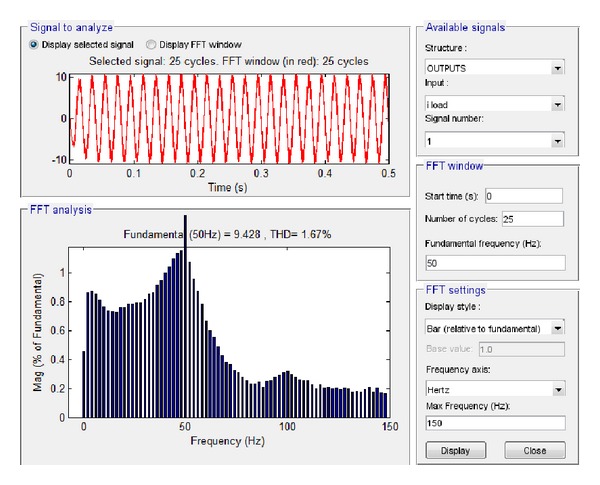
Current at load 1.5 KW with grid connection in Simulink.

First, the low load (1.5 KW) case was examined, starting with the output voltage at load and current at load. The subsequent simulation results via Simulink are shown in Figures 21 and 22, respectively.

Based on these plots it was found that the total harmonic distortion at load is 1.67%, while the amplitude of the voltage at load at the fundamental frequency is 331.9 V and the amplitude of the current at the fundamental frequency is 9.428 A.

**Figure 23:**
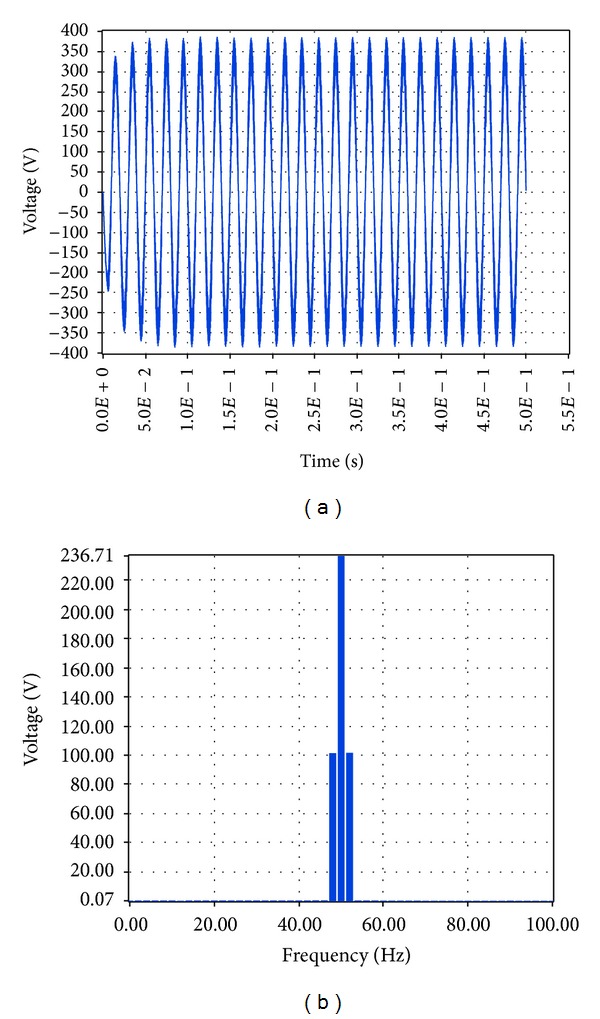
Voltage at load 1.5 KW with grid connection in LabVIEW.

**Figure 24:**
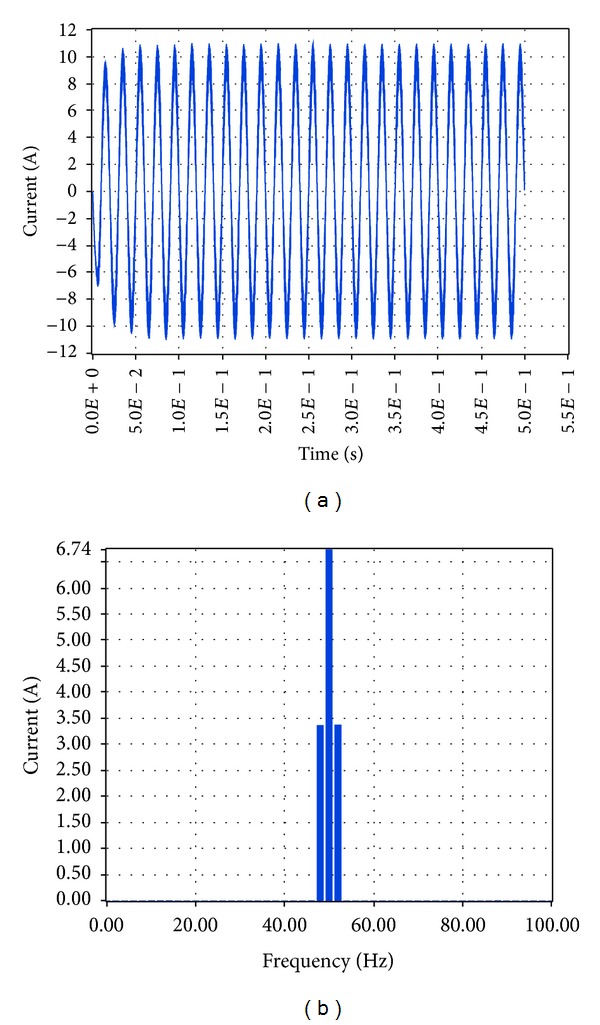
Current at load 1.5 KW with grid connection in LabVIEW.

The corresponding simulation results in LabVIEW proved that the amplitude of the voltage at the fundamental frequency is 236.71 V RMS, that is, 334.76 V, and the amplitude of the current at the fundamental frequency is 6.74 A RMS, that is, 9.53 A, while the total harmonic distortion at load is 11.25% as shown correspondingly in Figures 23 and 24.

**Figure 25:**
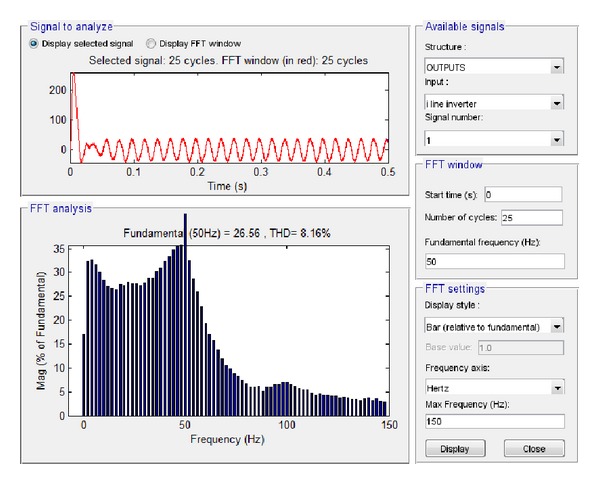
Current to load 1.5 KW and network with grid connection in Simulink.

**Figure 26:**
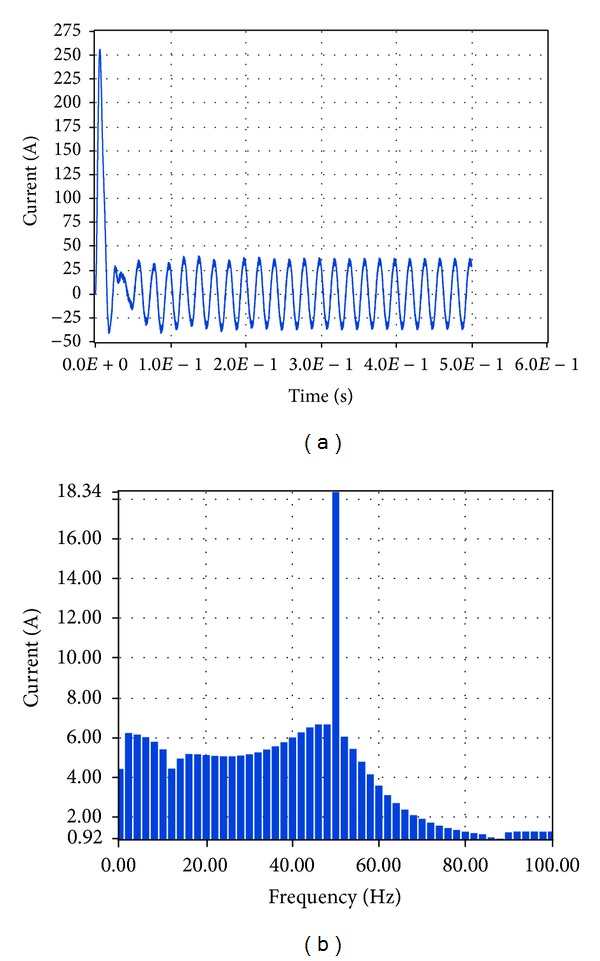
Current to load 1.5 KW and network with grid connection in LabVIEW.

Similarly, the total current at the connection of the system to the load and network was investigated. The resultant simulation plots drawn through the alternative utilization of Simulink and LabVIEW are depicted in Figures 25 and 26, respectively.

Based on these plots it was found that according to Simulink the amplitude of the total current at the connection of the system to the load and network is equal to 26.56 A while the total harmonic distortion is equal to 8.16%. The corresponding simulation results in LabVIEW proved that the current amplitude is equivalent to 18.34 A RMS, that is, 25.94 A, while THD is 6.65%.

**Figure 27:**
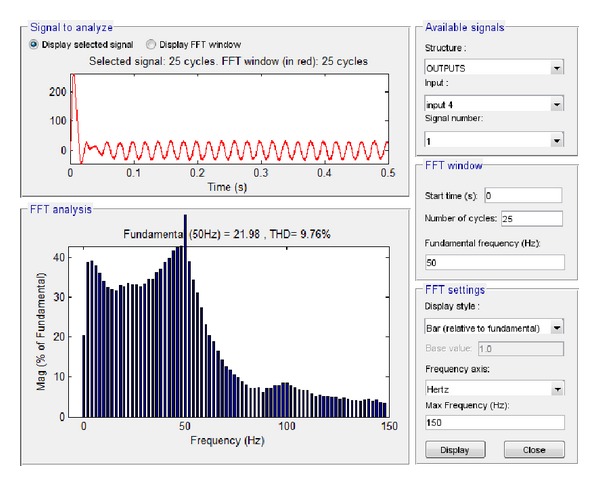
Current to network with grid connection and load 1.5 KW in Simulink.

**Figure 28:**
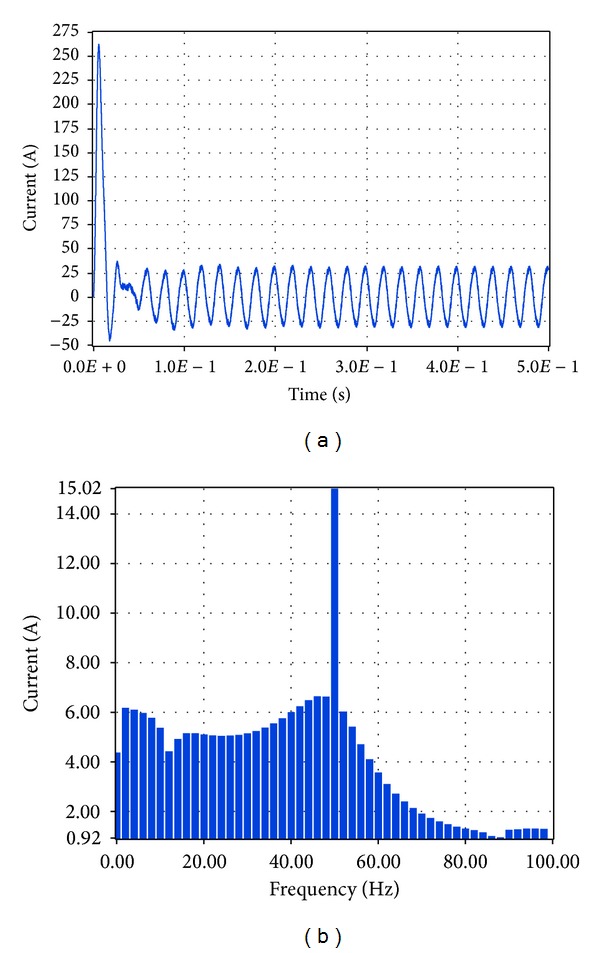
Current to network with grid connection and load 1.5 KW in LabVIEW.

Similarly, the current at the connection of the system to the network was examined. The resultant simulation plots via Simulink and LabVIEW are depicted in Figures 27 and 28, respectively. Based on these plots it was found that according to Simulink the amplitude of the total current at the connection to the network is equal to 21.98 A while the total harmonic distortion is equal to 9.76%. The corresponding simulation results in LabVIEW proved that the current amplitude is equivalent to 15.02 A RMS, that is, 21.24 A, while THD is 6.84%.

The comparative examination of the aforementioned simulation results regarding the 1.5 KW load case shows that the percentage deviation between Simulink and LabVIEW is equal to 0.85% for the voltage at load, 1.07% for the current at load, 2.33% for the total current to load and network, and 3.44% for the current to network.

**Figure 29:**
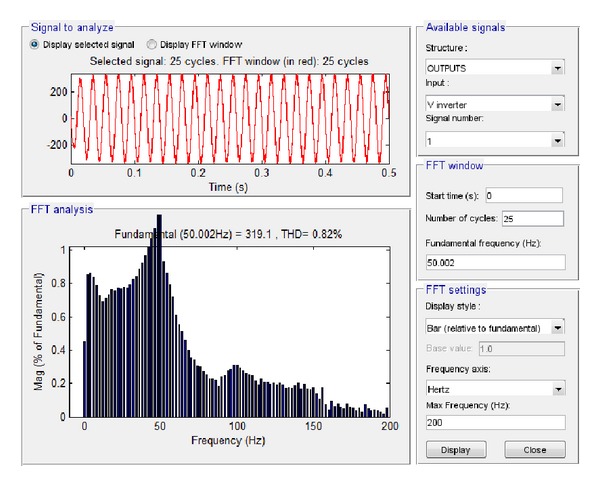
Voltage at load 6.5 KW with grid connection in Simulink.

**Figure 30:**
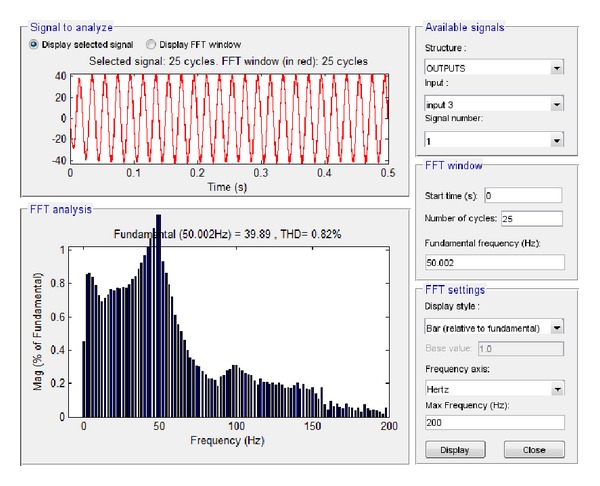
Current at load 6.5 KW with grid connection in Simulink.

Finally, the high load (6.5 KW) case was examined, starting with the output voltage at load and current at load. The subsequent simulation results via Simulink are shown in Figures 29 and 30, respectively.

**Figure 31:**
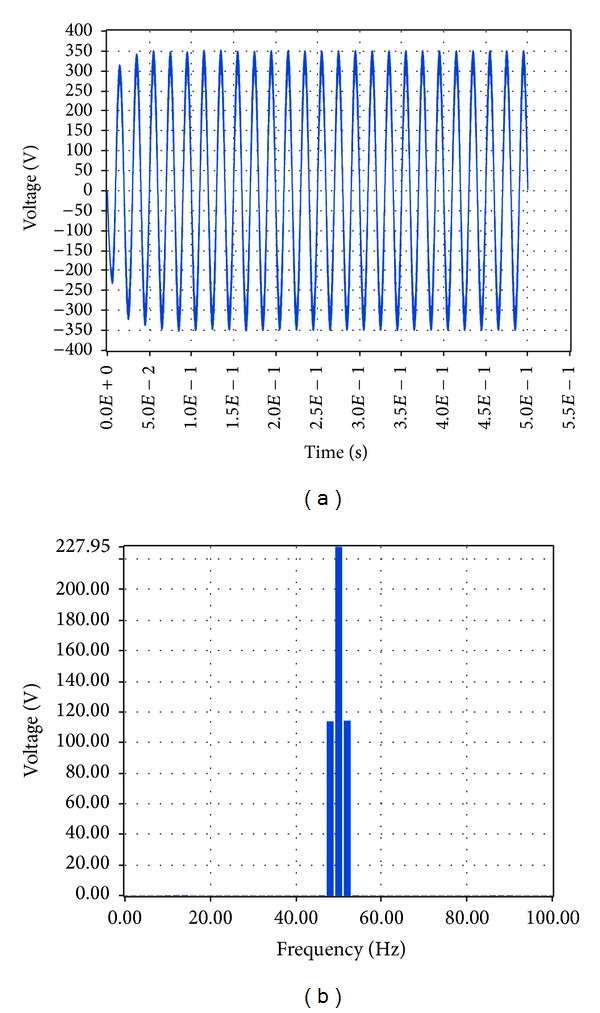
Voltage at load 6.5 KW with grid connection in LabVIEW.

**Figure 32:**
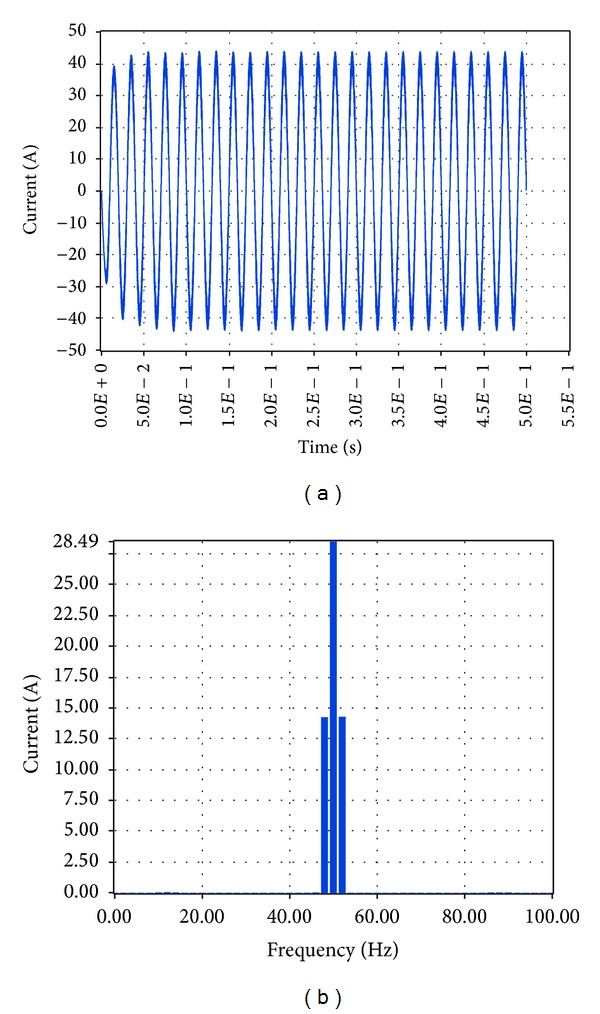
Current at load 6.5 KW with grid connection in LabVIEW.

Based on these plots it was found that the amplitude of the voltage at load at the fundamental frequency is 319.1 V, and the amplitude of the current at the fundamental frequency is 39.89 A, while the total harmonic distortion at load is 0.82%. The equivalent results in LabVIEW proved that the amplitude of the voltage at the fundamental frequency is 227.95 V RMS, that is, 322.37 V, and the amplitude of the current at the fundamental frequency is 28.49 A RMS, that is, 40.29 A while the total harmonic distortion at load is 7.83%, as shown correspondingly in Figures 31 and 32.

**Figure 33:**
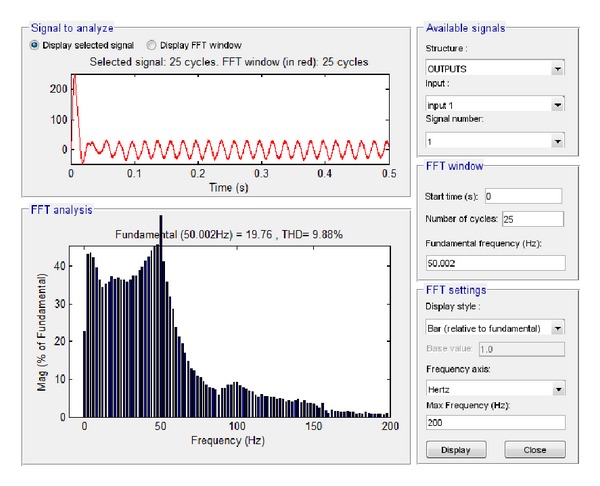
Current to load 6.5 KW and network with grid connection in Simulink.

**Figure 34:**
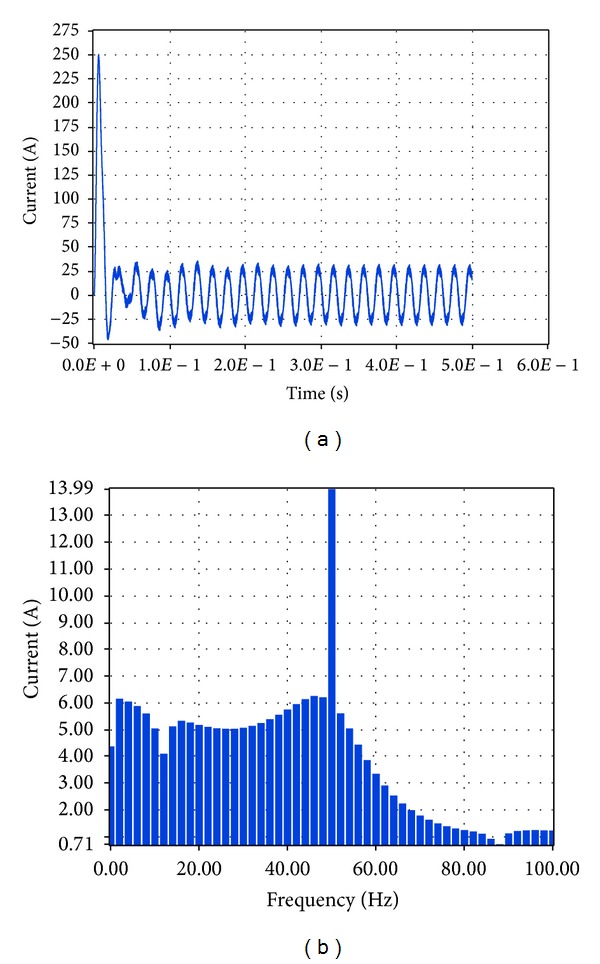
Current to load 6.5 KW and network with grid connection in LabVIEW.

Similarly, the total current at the connection of the system to the load and network was investigated. The resultant simulation plots via Simulink and LabVIEW are depicted in Figures 33 and 34, respectively.

Based on these plots it was found that according to Simulink the amplitude of the total current at the connection of the system to the load and network is equal to 19.76 A while the total harmonic distortion is equal to 9.88%. The corresponding simulation results in LabVIEW proved that the current amplitude is equivalent to 13.99 A RMS, that is, 19.78 A, while THD is 11.54%.

**Figure 35:**
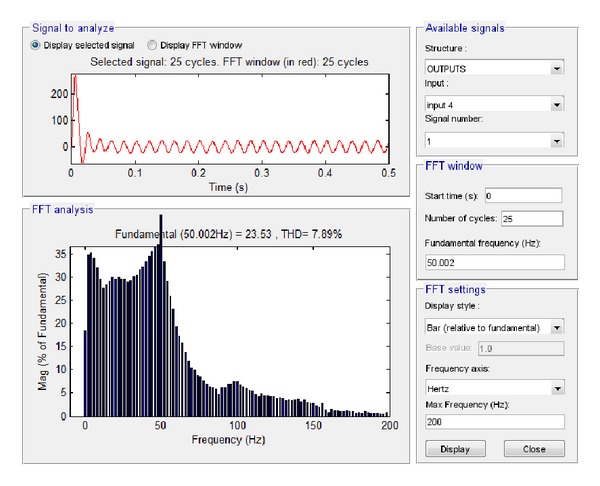
Current to network with grid connection and load 6.5 KW in Simulink.

**Figure 36:**
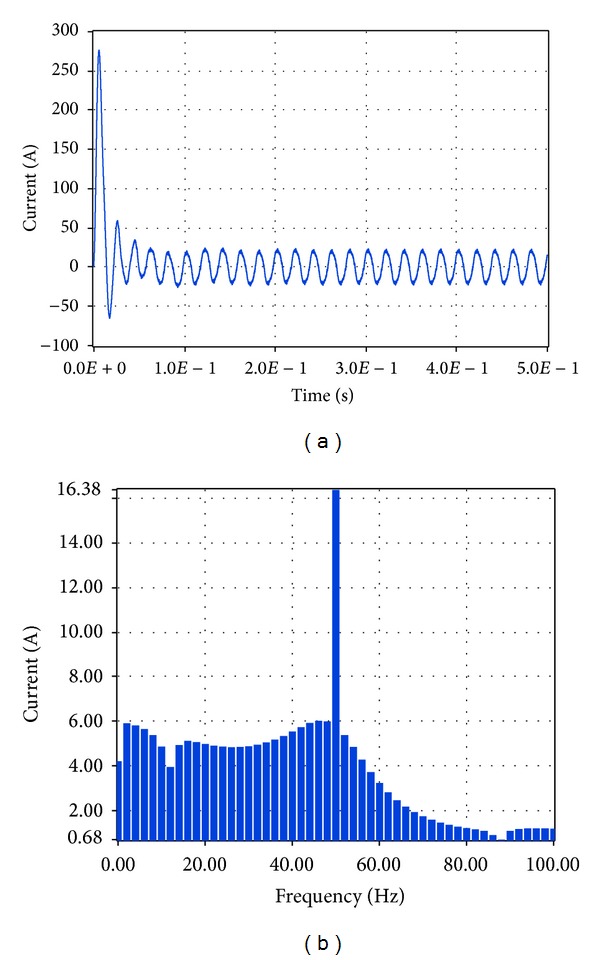
Current to network with grid connection and load 6.5 KW in LabVIEW.

Similarly, the current at the connection of the system to the network was examined. The resultant simulation plots via Simulink and LabVIEW are depicted in Figures 35 and 36, respectively.

Based on these plots it was found that according to Simulink the amplitude of the total current at the connection to the network is equal to 23.53 A while the total harmonic distortion is equal to 7.89%. The corresponding simulation results in LabVIEW proved that the current amplitude is equivalent to 16.38 A, RMS that is, 23.16 A, while THD is 10.4%.

The comparative examination of the aforementioned simulation results regarding the 6.5 KW load case shows that the percentage deviation between Simulink and LabVIEW is equal to 1.0% for the voltage at load, 1.0% for the current at load, 0.1% for the total current to load and network, and 1.57% for the current to network.

The correlation between the results attained via Simulink and LabVIEW for the performance simulation of the grid-connected integrated PV system can be synoptically evaluated in terms of the coefficient of determination (*R* ^2^). For this reason *R* ^2^ was computed for both the low load and high load cases by taking into consideration the values of all current amplitudes which were calculated.

**Figure 37:**
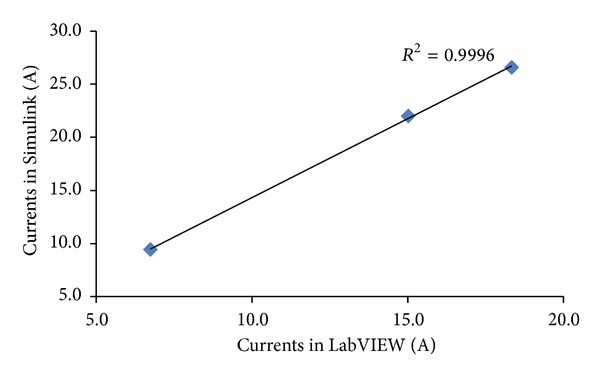
Coefficient of determination *R* ^2^ for the 1.5 KW load case.

**Figure 38:**
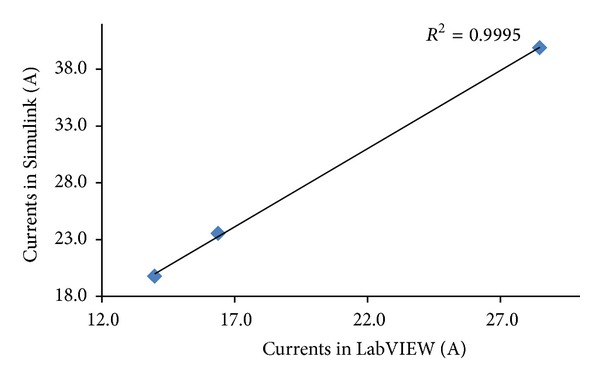
Coefficient of determination *R* ^2^ for the 6.5 KW load case.

Specifically, in the 1.5 KW case *R* ^2^ was found to be equal to 0.999 as shown in Figure 37. Similarly, in the 6.5 KW case *R* ^2^ was found to be also equal to 0.999 as illustrated in Figure 38.

## 6. Conclusions

The work presented in this paper focused on computer-aided model development and performance analysis of photovoltaic systems. For this reason, suitable models were built for various formations of photovoltaic schemes. All tasks performed were based on the use of two advanced graphical programming environments, namely, Simulink and LabVIEW. In this way, a comparative evaluation of the credibility of these software platforms was carried out.

Specifically, the case of a commercially available PV panel was studied. A model representing this panel was built and its operation was simulated. The simulation results regarding its characteristic features were compared to those provided by the PV panel manufacturer. The results of this comparison were positive in terms of both the modelling performed and the convergence between the outcomes of the two programming environments.

Next, the model of an integrated PV system having no grid connection was built and the performance analysis of this system was carried out by applying fast Fourier transformation. The simulation tests performed showed that both software platforms provide results which are almost the same regarding the electric entities simulated and slightly different regarding the signal total harmonic distortion.

Finally, the case of an integrated photovoltaic system connected to low-voltage grid was examined. Once again the model development was followed by the overall system performance analysis by examining the cases having a relatively low load and alternatively a higher load connected to the network. The application of fast Fourier transformation provided again results which have a small deviation regarding the electric entities simulated and a bigger one regarding the total harmonic distortion of the signals, thus indicating the existence of different noise influence.

## Figures and Tables

**Table 1:** Technical features of SX-50 PV panel.

Feature	Value
Maximum power (*P*_max⁡_)	50 W
Voltage at *P*_max⁡_ (*V*_max⁡_)	16.8 V
Current at *P*_max⁡_ (*I*_max⁡_)	2.97 A
Guaranteed minimum *P*_max⁡_	45 W
Short-circuit current (*I*_sc_)	3.23 A
Temperature coefficient of *I*_sc_	(0.065 ± 0.015)%/°C
Temperature coefficient of *V*_oc_	−(80 ± 10) mV/°C
Temperature coefficient of power	−(0.5 ± 0.05)%/°C

**Table 2:** Simulation results for *I*_max⁡_, *V*_max⁡_, and *P*_max⁡_ (*T* = 0°C).

	*I*_max⁡_ (A)	*V*_max⁡_ (V)	*P*_max⁡_ (W)
Simulink	2.995	18.226	54.607
LabVIEW	2.987	18.811	56.198

**Table 3:** Simulation results for *I*_max⁡_, *V*_max⁡_, and *P*_max⁡_ (*T* = 50°C).

	*I*_max⁡_ (A)	*V*_max⁡_ (V)	*P*_max⁡_ (W)
Simulink	3.002	14.916	44.782
LabVIEW	3.041	15.152	46.108

**Table 4:** Simulation results for *I*_max⁡_, *V*_max⁡_, and *P*_max⁡_ (*T* = 75°C).

	*I*_max⁡_ (A)	*V*_max⁡_ (V)	*P*_max⁡_ (W)
Simulink	2.998	13.024	38.925
LabVIEW	3.029	13.834	41.91

**Table 5:** Simulation results for *I*_max⁡_, *V*_max⁡_, and *P*_max⁡_ (*T* = 25°C).

	*I*_max⁡_ (A)	*V*_max⁡_ (V)	*P*_max⁡_ (W)
Simulink	2.998	16.86	50.55
LabVIEW	2.977	16.909	50.344
Solarex	2.97	16.8	50

**Table 6:** Percentage deviation between default values and simulation results for *I*_max⁡_, *V*_max⁡_, and *P*_max⁡_ (*T* = 25°C).

	*I*_max⁡_ (%)	*V*_max⁡_ (%)	*P*_max⁡_ (%)
Simulink	+0.951	+0.357	+1.1
LabVIEW	+0.247	+0.648	+0.688
